# Ecological Release and Venom Evolution of a Predatory Marine Snail at Easter Island

**DOI:** 10.1371/journal.pone.0005558

**Published:** 2009-05-20

**Authors:** Thomas F. Duda, Taehwan Lee

**Affiliations:** 1 Department of Ecology and Evolutionary Biology and Museum of Zoology, University of Michigan, Ann Arbor, Michigan, United States of America; 2 Smithsonian Tropical Research Institute, Balboa, Ancón, Republic of Panama; Northeastern University, United States of America

## Abstract

**Background:**

Ecological release is coupled with adaptive radiation and ecological diversification yet little is known about the molecular basis of phenotypic changes associated with this phenomenon. The venomous, predatory marine gastropod *Conus miliaris* has undergone ecological release and exhibits increased dietary breadth at Easter Island.

**Methodology/Principal Findings:**

We examined the extent of genetic differentiation of two genes expressed in the venom of *C. miliaris* among samples from Easter Island, American Samoa and Guam. The population from Easter Island exhibits unique frequencies of alleles that encode distinct peptides at both loci. Levels of divergence at these loci exceed observed levels of divergence observed at a mitochondrial gene region at Easter Island.

**Conclusions/Significance:**

Patterns of genetic variation at two genes expressed in the venom of this *C. miliaris* suggest that selection has operated at these genes and contributed to the divergence of venom composition at Easter Island. These results show that ecological release is associated with strong selection pressures that promote the evolution of new phenotypes.

## Introduction

Understanding the origins of biodiversity is a fundamental concern in biology. Ecological release, or the increased availability of resources afforded by reduced competition [1], can instigate adaptive radiations and was likely responsible for some of the most dramatic diversifications of life in earth's history [2], [3]. Ecological release seemingly promotes increased phenotypic variance and reduced stabilizing selection pressures due to a lower intensity of interspecific competition [4]. Although recent analyses support the prediction of greater niche variation in generalist populations that have experienced ecological release [5]–[7], with few exceptions [8], [9] very little is known about the heritability of this variation or the molecular genetic bases of the evolution of new phenotypes that result.

Members of the predatory marine gastropod genus *Conus* use a venom comprised of a diversity of ‘conopeptides’ or ‘conotoxins’ to paralyze their prey. *Conus* species also exhibit interspecific differences in both venom composition [10], [11] and feeding specialization [12], [13] that together suggest that species' venoms have evolved to most effectively paralyze their particular prey. To illuminate the factors that drive the evolution of *Conus* venoms and the impact of ecological release on venom evolution, we investigated the differentiation of venoms among populations of *Conus miliaris*, a species that shows evidence of dietary breadth expansion at Easter Island [14].

**Figure 1:**
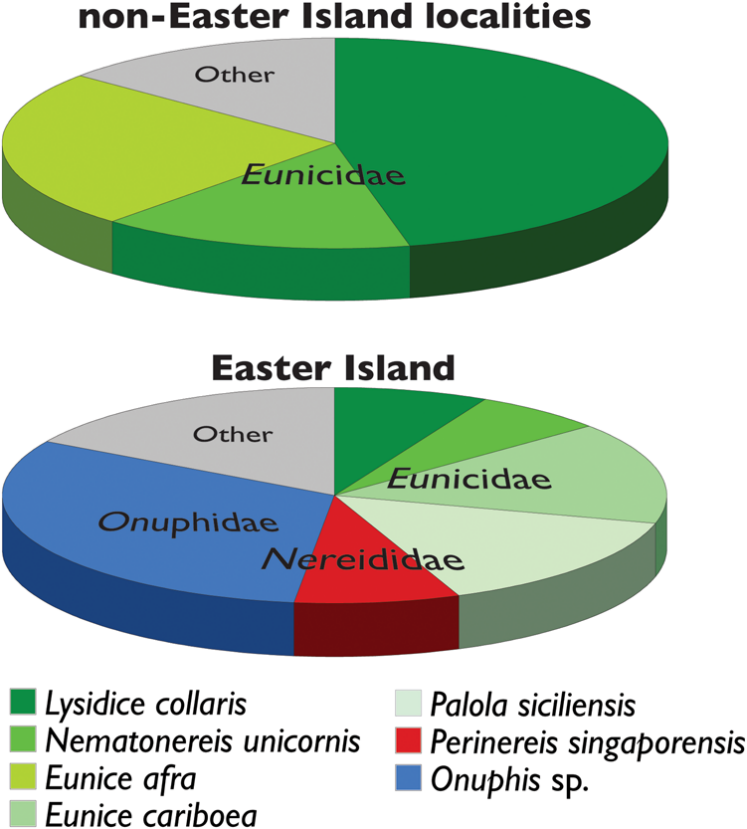
Prey items recovered from gut contents or feces of individuals of *Conus miliaris* at combined localities other than Easter Island (Seychelles, Maldive Islands, Great Barrier Reef, Marianas Islands, Marshall Islands and Caroline Islands) (*n* = 282) and at Easter Island (*n* = 310) as reported by Kohn (1978). Eunicidae includes *Lysidice collaris*, *Nematonereis unicornis*, *Eunice afra*, *Eunice cariboea* and *Palola siciliensis*; Nereididae includes *Perinereis singaporensis*; Onuphidae includes *Onuphis* sp.; ‘Other’ category includes additional prey species that were recovered at frequencies less than 5%.

Similar to several other widespread *Conus* species, *C. miliaris* occurs in tropical to subtropical shallow water environments from the Red Sea and eastern shores of Africa in the western Indian Ocean to Easter Island and Sala y Gómez in the southeastern Pacific [15]. Although other *Conus* are occasionally found at Easter Island, they are quite rare and only *C. miliaris* is common and abundant at this site [14], [16]. Presumably in response to the relative absence of congeners at Easter Island, *C. miliaris* has undergone ecological release: it preys on a more diverse assemblage of prey at Easter Island and is more abundant at Easter Island than at other localities in its range [14]. *C. miliaris* from most areas in the Indo-West Pacific, where it co-occurs with as many as 36 congeners [13], preys almost exclusively on three species of eunicid polychaetes (Fig. 1). But at Easter Island its diet is considerably broader and includes additional species of eunicids as well as several species of nereids, an onuphid and members of seven other polychaete families (Fig. 1).

The distinct ecology of *C. miliaris* at Easter Island offers a unique natural experiment to investigate the evolution of genes associated with ecological release. We specifically examined patterns of variation at two conotoxin loci among populations from Easter Island in the southeastern Pacific, Guam in the western Pacific, and American Samoa in the South Pacific and compared these to patterns of variation observed at a region of the mitochondrial cytochrome oxidase I (*COI*) gene.

## Methods

We obtained specimens from field collections at Hanga Roa, Easter Island and Pago Bay, Guam. Specimens from American Samoa and additional specimens from Guam were obtained from collections at the Florida Museum of Natural History (University of Florida, Gainesville, Florida, USA) and the University of Michigan Museum of Zoology. We extracted genomic DNA (gDNA) from 20–60 individuals from Easter Island, American Samoa and Guam using the E.Z.N.A. Mollusc DNA kit (Omega Bio-Tek). We also constructed venom duct cDNA from 18–20 individuals of *C. miliaris* from these locations as reported previously [17].

To assess the overall diversity of expressed conotoxin genes at different locations in the Pacific, we identified O-superfamily genes from cDNA of specimens from Easter Island and Guam by sequencing cloned amplification products obtained using general O-superfamily conotoxin primers TOX1 and TOX2 [17]. O-superfamily conotoxin genes encode a large class of peptides that block calcium, potassium and sodium channels [11] and 12 O-superfamily loci were previously identified from individuals of *C. miliaris* from American Samoa [10]. We identified 14 putative loci from individuals from Easter Island and Guam, and most matched previously identified loci of individuals of *C. miliaris* from American Samoa [10]. Two of these loci, *MIL2* and *MIL3*, appeared to have alleles that segregated geographically and that encoded unique conotoxin peptides. Sequences of other putative loci were from too few individuals or locations to ascertain allelic segregation patterns or in some cases segregation patterns did not appear as strong as those observed at *MIL2* and *MIL3*. We designed locus-specific primers for the *MIL2* and *MIL3* loci (MIL2C: CAAAAACTCCAAGATGACCAGGGAT and MIL3E: CAAAAACTCCAGGATGACCAKGGGT) and used them individually with TOX2 to assay *MIL2* and *MIL3* genotypes of individuals from Easter Island, Guam and American Samoa. Both sets of primers amplify 132 bp, including 84 bp of the mature conotoxin coding region and 48 bp of the 3′ untranslated region. Based on our inability to amplify particular regions of O-superfamily conotoxins from gDNA, O-superfamily loci apparently contain a large intron approximately 30 bp upstream of the mature toxin coding region. Thus the locus-specific primers were designed downstream of this suspected intron position to permit amplification of these gene sequences from gDNA. We attempted amplifications from cDNA and gDNA of specimens from all three locations. We directly sequenced amplification products and identified alleles of heterozygotes based on presence of double peaks in chromatograms and by comparing these sequences to confirmed sequences of alleles that were determined from cloning or from direct sequencing of amplification products of putative homozygotes. We cloned products that contained sequences of more than one locus. Sequences from other putative loci were occasionally amplified when using cDNA as a template; this presumably resulted from non-specific priming for individuals that did not express or that weakly expressed *MIL2* or *MIL3*. We also sequenced templates with allele-specific primers for heterozygous individuals with unique alleles (i.e., the allele with the unique base substitution could not be determined).

We examined chromatograms and aligned sequences using Sequencher version 4.8 (Gene Codes Corporation). We used TCS version 1.21 [18] to construct a statistical parsimony network [19] for *MIL2* and *MIL3* sequences and for published mitochondrial *COI* sequences of *C. miliaris* from Guam (*n* = 19), American Samoa (*n* = 31) and Easter Island (*n* = 61) [20] (GenBank accession numbers FJ392914–FJ392994, FJ411486–411515). We estimated F-statistics and conducted an analysis of molecular variance (AMOVA) for these three loci with Arlequin version 2.000 [21]. We used Kimura 2-parameter distances [22] in computations of *Φ*_ST_ values for the *MIL2* locus, Jukes Cantor distances [23] for the *MIL3* locus, and Tamura-Nei distances [24] for the *COI* gene, based on the most appropriate model of nucleotide substitution for each gene as determined with Modeltest version 3.7 [25]. We calculated the proportions of nonsynonymous substitutions (*d*_N_) and synonymous substitutions (*d*_S_) per respective site among alleles of the *MIL2* and *MIL3* loci with a maximum likelihood approach employed in PAML version 3.15 [26]. Because only few substitutions were observed among alleles and only 84 bp of the mature toxin coding region were examined, we did not conduct formal tests of positive selection.

## Results and Discussion

Using locus-specific amplifications of conotoxin loci *MIL2* and *MIL3*, we identified genotypes of multiple specimens of *C. miliaris* from Easter Island (*n* = 54 for *MIL2*; *n* = 46 for *MIL3*), American Samoa (*n* = 24 for *MIL2*; *n* = 16 for *MIL3*) and Guam (*n* = 21 for *MIL2*; *n* = 18 for *MIL3*) from amplifications of cDNA and/or gDNA. We were unable to recover sequences of *MIL2* and *MIL3* from cDNA of some of the individuals examined (which suggests that these genes may not be expressed by all individuals), but in most cases we were able to obtain sequences from these specimens with amplifications of gDNA. Segregation patterns of unique sequences (i.e., putative alleles) of both loci within and between individuals strongly imply that these sequences represent alleles of single loci and are not alleles of recently duplicated genes. In particular, all locus-specific amplifications from gDNA and most from cDNA (see above) yielded only one or two unique sequences from each individual; we never observed an individual that contained three or more putative alleles. We did not detect any evidence of inter-allelic recombination at either locus (i.e., we never observed a mosaic sequence that was comprised of segments of two putative alleles). Nonetheless, because we examined chromatograms that were obtained from direct sequencing of amplification products, inter-allelic recombination events would not be apparent in heterozygotes.

**Figure 2:**
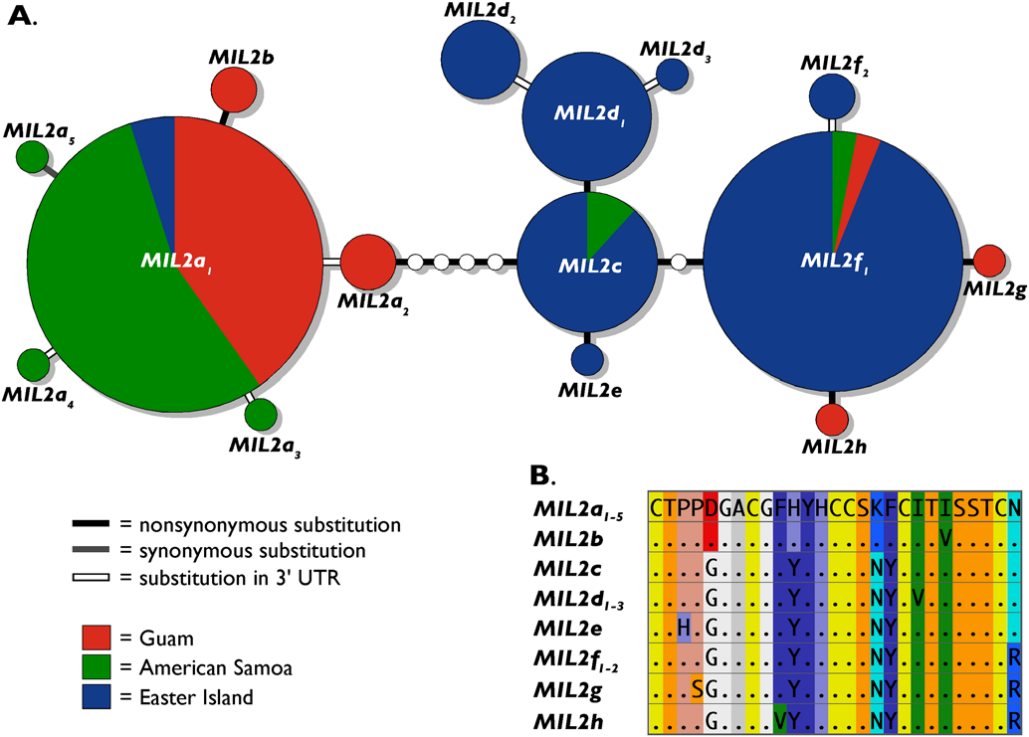
Allelic diversity of *Conus miliaris* conotoxin locus *MIL2*. A. Haplotype network of alleles of conotoxin locus *MIL2* of *Conus miliaris* at Guam, American Samoa and Easter Island. Haplotypes are illustrated as circles; hypothetical haplotypes that were not observed are illustrated as small, empty circles. Areas of circles are proportional to frequencies of alleles; pie diagrams illustrate the allelic frequencies at each location. Substitution types are shown as indicated in figure. Names of alleles were assigned based on distinctiveness of translated amino acid sequences of alleles; sets of alleles with identical amino acid translations (but different nucleotide sequences) are distinguished by numeral subscripts. B. Predicted amino acid sequences of alleles of locus *MIL2*. Amino acids are provided as single letter codes. To illustrate radical amino acid substitutions, amino acids with similar properties are provided in the same background color (coloring based on the ‘amino’ scheme utilized in Jmol, a Java viewer for chemical structures (http://www.jmol.org/)). Amino acid sequences of sets of alleles that exhibit no nonsynonymous substitutions (*MIL2a_1_*_–*5*_, *MIL2d_1_*_–*3*_ and *MIL2f_1_*_–*2*_) were merged.

We recovered 15 alleles from locus *MIL2*: three that occurred at two or three localities and 12 that were unique to a single location (Fig. 2A) (GenBank accession numbers FJ613506–FJ613520). These alleles differ at 1–11 base pairs (bp) (Fig. 2A). Only one allele (*MIL2a_5_*) is distinguished by a synonymous substitution within the toxin coding region of the gene (Fig. 2A). Six other alleles exhibit single substitutions within the 3′ untranslated region. The remaining eight alleles differ only at nonsynonymous sites that are responsible for one to seven, mostly nonconservative amino acid substitutions (i.e., charge or polarity altering substitutions) among the 28 amino acids of the translated conotoxin peptides (Figs. 2A, 2B; Table S1).

**Figure 3 pone-0005558-g003:**
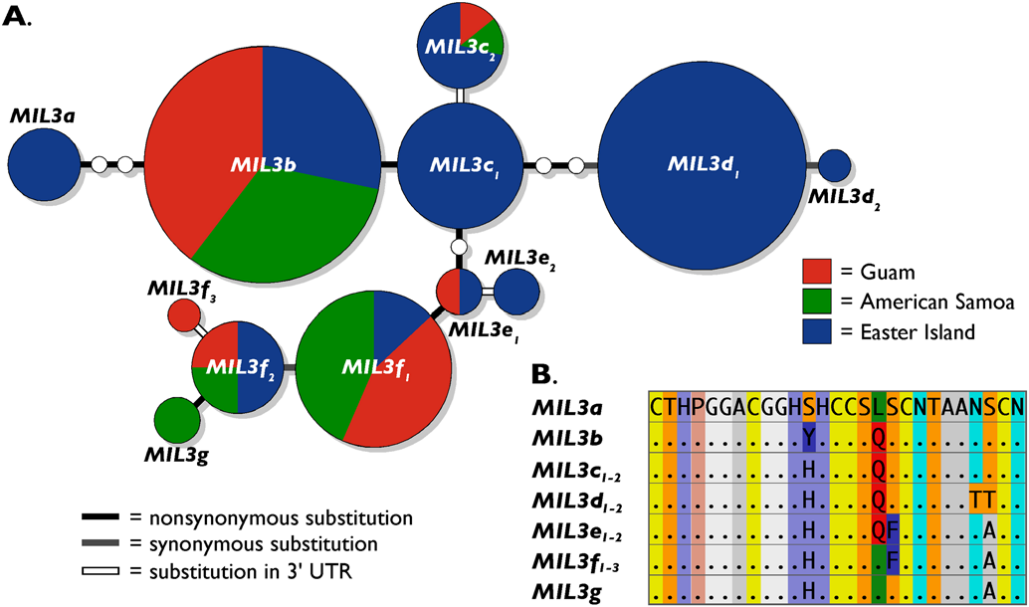
Allelic diversity of *Conus miliaris* conotoxin locus *MIL3*. A. Haplotype network of alleles of conotoxin locus *MIL3* of *Conus miliaris* at Guam, American Samoa and Easter Island. Haplotypes are illustrated as described in Fig. 2. Substitution types are shown as indicated in figure. Names of alleles were assigned as described in Fig. 2. B. Predicted amino acid sequences of alleles of locus *MIL3*. Amino acids are provided as single letter codes. We used the same amino acid coloring scheme as described in Fig. 2. Amino acid sequences of sets of alleles that exhibit no nonsynonymous substitutions (*MIL2c_1_*_–*2*_, *MIL2d_1_*_–*2*_, *MIL2e_1_*_–*2*_ and *MIL2f_1_*_–*3*_) were merged.

We recovered 12 *MIL3* alleles, including seven that were unique to a single locality (Fig. 3A) (GenBank accession numbers FJ716816–FJ716827). While most sequences differed at nonsynonymous sites, two alleles (*MIL3c_2_* and *MIL3e_2_*) exhibited single substitutions within the 3′ untranslated region and other alleles exhibited synonymous substitutions within the toxin coding region (Figs. 3A, 3B; Table S2). As with *MIL2*, most amino acid substitutions are nonconservative.

Seven of the *MIL2* alleles and five of the *MIL3* alleles are restricted completely or almost exclusively to Easter Island, while another eight *MIL2* alleles and two *MIL3* alleles are unique or nearly exclusive at American Samoa and/or Guam (Figs. 2A and 3A). Based on analyses of F-statistics, *C. miliaris* at American Samoa and Guam show no evidence of population structure at *MIL2* and *MIL3* (Table 1). A single *MIL2* allele (*MIL2a_1_*) occurs at frequencies of 0.854 and 0.786 and the two common *MIL3* alleles (*MIL3b* and *MIL3f_1_*) occur at similar frequencies (0.531 and 0.583, 0.313 and 0.278 respectively) at American Samoa and Guam. Except for *MIL3b*, the common alleles at American Samoa and Guam are present at only low frequency at Easter Island (*MIL2a_1_*: 0.037; *MIL3b*: 0.163; *MIL3f_1_*: 0.033). Instead three other alleles at *MIL2* (*MIL2c*, *MIL2d_1_* and *MIL2f_1_*) predominate here with a combined frequency of 0.870. These three alleles are either absent or rare at American Samoa and Guam. Also, one of the *MIL3* alleles that is absent at American Samoa and Guam (*MIL3d_1_*) is the most common allele at Easter Island with an observed frequency of 0.446. As expected from these patterns, F-statistics show that *C. miliaris* at Easter Island is genetically differentiated at *MIL2* and *MIL3* from *C. miliaris* at American Samoa and Guam with large and highly significant pairwise *Φ*_ST_ values estimated between these locations (Table 1). Moreover, approximately 75.5% of the genetic variance at *MIL2* and 25.9% of the variance at *MIL3* are partitioned among Easter Island and combined American Samoa and Guam samples. Thus, while *C. miliaris* at Guam and American Samoa show no genetic differentiation at conotoxin loci *MIL2* and *MIL3*, the population at Easter Island remarkably exhibits significantly different allelic frequencies at these loci.

**Table 1:** Pairwise *Φ*_ST_ values among populations of *Conus miliaris* estimated from analysis of sequences of conotoxin loci *MIL2* and *MIL3* and mitochondrial *COI* sequence data.

	*MIL2*	*MIL3*	*COI*
Easter Island - American Samoa	0.732**	0.235**	0.143*
Easter Island - Guam	0.761**	0.236**	0.121*
American Samoa - Guam	−0.049^NS^	−0.024^NS^	−0.012^NS^

Probabilities that observed *Φ*_ST_ values deviate from a null hypothesis of no difference between populations were determined from the proportion of 10,100 permutations of haplotypes between populations that gave *Φ*_ST_ values greater than or equal to the observed *Φ*_ST_ (NS = not significant, * = *P*<0.001, ** = *P*<0.0001).

**Figure 4:**
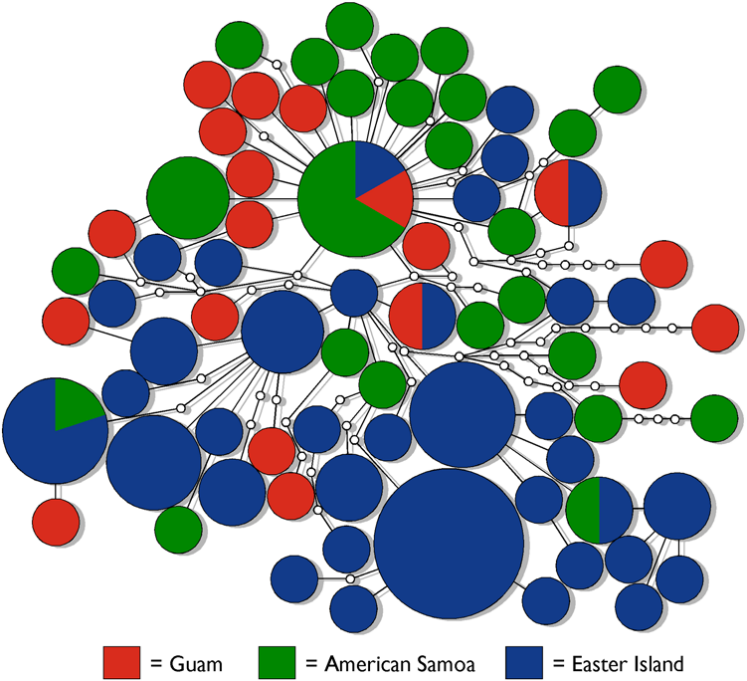
Haplotype network of *COI* sequences from 111 individuals of *Conus miliaris* at Guam, American Samoa and Easter Island. Haplotypes are illustrated as circles, with hypothetical haplotypes that were not observed illustrated as small, empty circles. Areas of circles are proportional to the haplotype frequencies; pie diagrams illustrate frequencies of haplotypes at each location.

The observed genetic differentiation of *C. miliaris* at Easter Island could be related to the geographic isolation of *C. miliaris* at Easter Island, the world's most isolated oceanic island, or associated with the increased dietary breadth of the Easter Island population. Several lines of evidence support the latter hypothesis. If genetic drift or other demographic phenomena correlated with isolation at Easter Island were solely responsible for the significantly different allelic frequencies of *MIL2* and *MIL3* at this location, we expect that levels of divergence at these loci would be comparable and that other loci would show similar patterns. Indeed, detection of outlier *F*_ST_ values is an effective strategy for identifying loci under selection [27]–[29]. But *MIL2* shows much stronger divergence than *MIL3* based on pairwise *Φ*_ST_ estimates (Table 1) and AMOVA results. The rate of fixation of mitochondrial genes is four times greater on average than the rate of fixation of nuclear autosomal genes; this is because the effective population size of mitochondrial loci is one-fourth that of nuclear autosomal loci due to the haploidy and uniparental inheritance of the mitochondrial genome and diploidy and biparental inheritance of the nuclear genome [30]. Thus, we expect mitochondrial genes to show much more differentiation at Easter Island than observed at *MIL2* and *MIL3* if patterns of divergence at these conotoxin loci are solely a result of genetic drift. Examination of mitochondrial *COI* sequences of *C. miliaris* at Easter Island, American Samoa and Guam, however, reveals that although *C. miliaris* at Easter Island is genetically differentiated from the other locations, the level of divergence observed at *COI* is much less than at *MIL2* and *MIL3* (Table 1, Fig. 4). In addition, while 75.6% and 25.9% of the genetic variance at *MIL2* and *MIL3* respectively are partitioned among Easter Island and combined samples from American Samoa and Guam, only 13.8% of the variance at *COI* is partitioned among these locations.

Although *COI* haplotypic diversity of *C. miliaris* is high and the extent of genetic differentiation can be underestimated with data from highly variable genetic loci (e.g., microsatellites) [31], the geographic distribution of *COI* haplotypes represented in the haplotype network is in clear stark contrast to the geographic distribution of *MIL2* and *MIL3* alleles (Fig. 2–4). In other words, if genetic drift were solely responsible for the observed levels of genetic differentiation at *MIL2* and *MIL3*, *COI* sequences should exhibit geographic structure regardless of the extent of the haplotypic diversity of this locus.

The low level of divergence of *COI* at Easter Island could also be explained by a recent selective sweep of the mitochondrial genome, but the high haplotypic diversity at *COI* suggests this is not the case. Also, previous investigation of the demography of *C. miliaris* based on analyses of *COI* sequences gives a time of expansion of Pacific populations (or the time of a selective sweep of the mitochondrial genome if one had occurred) of 0.68 million years (95% confidence interval: 0.42 to 1.36 million years) and time of separation of the Easter Island population at 0.45 million years ago (confidence interval: 0.32–0.67 million years ago) [20]. Although the 95% confidence intervals of these events overlap, the estimates of the timing of these events suggests that if there had been a selective sweep of the mitochondrial genome, it occurred prior to or coincident with the founding of the Easter Island population.

These results are hence contrary to the expectation that *C. miliaris* would exhibit more structure at the mitochondrial locus than the nuclear ones and suggest that the isolation of the Easter Island population (i.e., genetic drift) alone cannot explain the observed level of divergence at *MIL2* or *MIL3*. Instead, both conotoxin loci have likely been subject to directional or disruptive selection that has apparently been stronger at *MIL2* based on the greater divergence observed at this locus. Nonetheless, data from additional loci, especially neutral markers from the nuclear genome as well as other conotoxin genes, and samples from additional locations would certainly aid in more thoroughly testing these hypotheses and confirming our interpretations.

The patterns of divergence at *MIL2* and *MIL3* instead compares well with the divergent feeding ecology of *C. miliaris* at Easter Island. The increased dietary breadth at this isolated location appears to be associated with selection for distinct *MIL2* and *MIL3* allelic variants. These results suggest that strong selection pressures drive the evolution of new phenotypes in populations undergoing ecological release. Conotoxin peptides that differ at even one amino acid are functionally distinct [32] and so the patterns of genetic differentiation observed at *MIL2* and *MIL3* imply that the population of *C. miliaris* contains a different repertoire of venom components at Easter Island than at American Samoa and Guam. The gene products of these loci presumably represent only a small proportion of the expressed venom components in these populations. Because we do not yet know the patterns of variation at other expressed conotoxin loci, our results do not show that all venom components have diverged in this manner. Nonetheless, even if alleles of all other expressed conotoxin genes show complete homogeneity across the distribution of *C. miliaris*, this would not affect our interpretations about the strength of selection at *MIL2* and *MIL3*.

Ecological release can theoretically promote the evolution of novelty [33]. Our results suggest that broad dietary breadth drove the evolution of venom at Easter Island. We suspect that the gene products of the unique or more frequent alleles at this location are more effective at paralyzing a more diverse array of prey or are specific to prey that are uniquely only consumed at Easter Island. Clearly, functional studies of venoms and translated products of *MIL2* and *MIL3* alleles and especially their effects on prey as well as investigations of functions of gene products and patterns of variation of other conotoxin loci are needed to illuminate the bases of these selection pressures.

We anticipate that genes that influence ecological attributes of other species experiencing ecological release will exhibit a similar mode of evolution as observed for *MIL2* and *MIL3*. This should be especially apparent for the adaptive evolution of genes encoding venom components of other venomous taxa, as has been suggested for snakes [34]–[36] and spiders [37], but also for genes that affect morphological, physiological and behavioral aspects that are tightly linked with an organism's ecology in other species that have undergone ecological release.

## Supporting Information

Table S1 Proportions of nonsynonymous substitutions (*d*_N_) (below diagonal) and synonymous substitutions (*d*_S_) (above diagonal) per respective site among alleles of *Conus miliaris* conotoxin locus *MIL2* that exhibit substitutions within the mature toxin coding region.
(0.08 MB DOC)

Table S2 Proportions of nonsynonymous substitutions (*d*_N_) (below diagonal) and synonymous substitutions (*d*_S_) (above diagonal) per respective site among alleles of *Conus miliaris* conotoxin locus *MIL3* that exhibit substitutions within the mature toxin coding region.
(0.05 MB DOC)
