# Supplementary material for: Ecological Release and Venom Evolution of a Predatory Marine Snail at Easter Island
Source: PLoS One. 2009 May 20;4(5):e5558. doi: 10.1371/journal.pone.0005558 (PMC2680045; doi:10.1371/journal.pone.0005558)
Supplement: Table S2 — Proportions of nonsynonymous substitutions (d N) (below diagonal) and synonymous substitutions (d S) (above diagonal) per respective site among alleles of Conus miliaris conotoxin locus MIL3 that exhibit substitutions within the mature toxin coding region. (0.05 MB DOC) [file pone.0005558.s002.doc]

Supporting Information

**Table S2**. Proportions of nonsynonymous substitutions (*d*_N_) (below diagonal) and synonymous substitutions (*d*_S_) (above diagonal) per respective site among alleles of *Conus miliaris* conotoxin locus *MIL3* that exhibit substitutions within the mature toxin coding region.

|  | *MIL3a* | *MIL3b* | *MIL3c_1-2_* | *MIL3d_1_* | *MIL3d_2_* | *MIL3e_1-2_* | *MIL3f_1_* | *MIL3f_2-3_* | *MIL3g* |
| --- | --- | --- | --- | --- | --- | --- | --- | --- | --- |
| *MIL3a* |  | 0.000 | 0.001 | 0.056 | **0.108** | 0.001 | 0.001 | 0.044 | 0.048 |
| *MIL3b* | 0.032 |  | 0.000 | 0.050 | **0.097** | 0.001 | 0.001 | 0.046 | 0.050 |
| *MIL3c_1-2_* | 0.051 | 0.020 |  | **0.0636** | **0.114** | 0.000 | 0.001 | **0.052** | **0.066** |
| *MIL3d_1_* | 0.087 | 0.053 | **0.0321** |  | **0.034** | 0.041 | 0.045 | **0.091** | **0.096** |
| *MIL3d_2_* | **0.089** | **0.054** | **0.033** | **0.000** |  | **0.084** | **0.091** | **0.134** | **0.144** |
| *MIL3e_1-2_* | 0.089 | 0.056 | 0.034 | 0.058 | **0.058** |  | 0.000 | **0.058** | **0.045** |
| *MIL3f_1_* | 0.073 | 0.071 | 0.050 | 0.073 | **0.074** | 0.015 |  | **0.035** | **0.035** |
| *MIL3f_2-3_* | 0.074 | 0.072 | **0.051** | **0.074** | **0.075** | **0.016** | **0.000** |  | 0.000 |
| *MIL3g* | 0.053 | 0.052 | **0.032** | **0.053** | **0.054** | **0.036** | **0.019** | 0.019 |  |

Values of pairwise comparisons in which *d*_N_ is less than *d*_S_ are in bold.
